# Sonochemically prepared hierarchical MFI-type zeolites as active catalysts for catalytic ethanol dehydration

**DOI:** 10.1016/j.ultsonch.2021.105581

**Published:** 2021-05-03

**Authors:** Ł. Kuterasiński, U. Filek, M. Gackowski, M. Zimowska, M. Ruggiero-Mikołajczyk, P.J. Jodłowski

**Affiliations:** a Jerzy Haber Institute of Catalysis and Surface Chemistry, Polish Academy of Sciences, ul. Niezapominajek 8, 30-239 Cracow, Poland; b Faculty of Chemical Engineering and Technology, Cracow University of Technology, Warszawska 24, 31-155 Cracow, Poland

**Keywords:** Sonochemistry, Desilication, Mesoporosity, MFI-type zeolite, Catalyst, Ethanol dehydration

## Abstract

In this paper, the ultrasonic-assisted desilication technique was reported as an attractive and efficient way for the preparation of hierarchical zeolites with MFI structure type. The prepared materials were used as active catalysts for the dehydration of ethanol into diethyl ether and ethylene. For all catalysts, the selectivity to diethyl ether was ca 95% or higher up to 210 °C, with catalytic activity in the range of 40–68%. In case of desilicated zeolites, at 270–290 °C, the conversion of ethanol was full with selectivity to ethylene ca 80%. MFI-type commercial zeolite was treated with a sodium and/or tetrabutylammonium hydroxide aqueous solutions (NaOH or NaOH/TBAOH) for 30 min. In the case of the application of ultrasounds, a QSonica Q700 sonicator (60 W and 20 kHz) equipped with a “1” diameter horn was used. In all cases, desilication was performed in an ice bath in order to keep the procedure conditions at low temperature.

It was indicated that the use of ultrasounds during desilication procedure caused higher extraction of silicon and aluminum, which was connected with an elevated mesoporosity in relation to the samples modified in the absence of ultrasounds. Ultrasonic-assisted treatment of MFI-type zeolite caused also an apparent formation of numerous holes inside zeolite grains, resembling the look of “swiss cheese”. Furthermore, it was indicated that the samples prepared using ultrasonic irradiation exhibited enhanced catalytic properties in the dehydration of ethanol. For instance, MFI-type zeolite treated with NaOH/TBAOH alkaline mixture containing 10 mol% of TBAOH in the presence of ultrasounds (M−10 s) demonstrated higher both conversion of ethanol (59% vs. 47%) and selectivity to diethyl ether (95% vs. 93%) in comparison with zeolite modified conventionally (M−10c).

The best catalyst was zeolite ultrasonically desilicated with NaOH/TBAOH solution of 70 mol% of TBAOH (M-70s). Generally, this catalyst indicated the highest conversion of ethanol, very high selectivity to diethyl ether (94-100%) at 150-210  °C and the highest selectivity to ethylene among investigated catalysts (21%, 66% and 84%) at 230  °C, 250 ^o^C and 270  °C.

## Highlights

• Ultrasonic irradiation of zeolites enhances Si and Al extraction.
• Ultrasounds improves mesoporosity of prepared zeolites.
• Ultrasonic desilication changes neither the crystallinity nor morphology.
• Ultrasonic desilication improves catalytic properties of MFI-type zeolites.

## Graphical abstract

**Figure:**
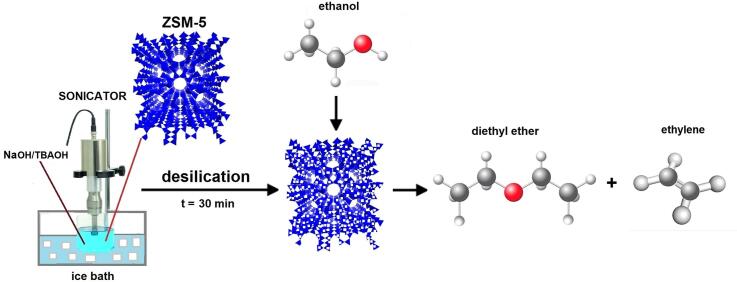

## 1 Introduction

Zeolites are crystalline and microporous materials built mainly of silicon, aluminum, and oxygen. The occurrence of Brønsted acid sites inside micropores allows to use this group of aluminosilicates as catalysts in numerous industrial processes [1], [2]. However, due to a limited accessibility of the active sites and slow transport of reactants in micropores, which can lead to rapid deactivation of catalyst, the application of this groups of minerals as catalysts is not fully satisfying [3], [4], [5]. The formation of mesopores via alkaline treatment of zeolites seems to resolve this problem [6], [7], [8].

One of the routes in the synthesis of mesoporous materials is the application of ultrasonic irradiation. This technique allows to reduce the duration procedure, the use of milder pressure and temperature conditions and may cause the limitation (or even elimination) of the utilization of expensive and toxic reagents, which can also result in the decrease of synthesis costs. The phenomenon of the replacement of conventional desilication of microporous zeolites (in alkaline media) with ultrasonic technique is based on the cavitation mechanism of ultrasounds propagation and the formation of local “spots” of ultrahigh temperature (5000 K) and pressure (1000 bar). The cavitation leads to collisions of particles moving at high velocities, promoting the formation of radicals triggering sonochemical reactions [9], [10].

The application of ultrasounds indicated beneficial effects in syntheses of zeolites: CHA [11], A [12], NaP [13], bilikalite [14], MCM-22 [15] and RHO-type zeolites [16]. Generally, ultrasonic-assisted synthesis of zeolites caused the generation of products in a shorter time, which presented an improved crystallinity degree and smaller crystal sizes.

Ultrasonic irradiation was also used in the preparation of single metal oxides, such as: ZrO_2_ [17], TiO_2_ [18], MnO_2_ [19], Cu_2_O [20], CeO_2_ [21] and ZnO [22]. Furthermore, ultrasonic-assisted procedure of the synthesis of mixed oxides was reported for: Ba_y_Zr_3-y_TiO_3_ [23], NiO/Al_2_O_3_ [24], Ce_0.5_Zr_0.5_O_2_ [25], CuO/ZnO/ZrO_2_/Al_2_O_3_ [26] and Ni-Co/Al_2_O_3_-ZrO_2_ [27].

Ultrasonic-assisted modification of zeolites was also reported in literature. Hosseini et al. [28] performed dealumination of zeolite Y in ethanol-acetylacetone solution as a chelating agent both in the absence and presence of ultrasounds. It was shown that the sonochemical-assisted modification of zeolite samples resulted in a higher aluminum extraction from zeolite framework than for the materials prepared conventionally.

Zhang et al. [29] obtained mesoporous FAU-type zeolites via chemical dealumination (using citric acid and H_4_EDTA aqueous solutions) and a subsequent ultrasonic-assisted alkaline treatment in aqueous NaOH solutions. The use of ultrasounds accelerated the formation of mesoporosity in respect to the analogues prepared traditionally. Similar observations were reported by Oruji et al*.* [30], who synthesized mesoporous FAU-type zeolites in sodium form. Based on porosity studies, it was shown that the rising duration of base-wash procedure led to gradual increase of mesoporosity with a simultaneous decrease of crystallinity.

In turn, Kuterasiński et al. [31] investigated the effect of ultrasonic-assisted desilication of commercial FAU-type zeolite. The prepared samples have been used as catalysts for the decarbonylation of furfural into furan. It was shown that the application of high-frequency ultrasounds during alkaline treatment procedure caused higher mesoporosity and enhanced catalytic properties in respect to the catalysts modified under conventional desilication conditions.

Khoshbin and Karimzadeh [32] prepared mesoporous ZSM-5 zeolite via ultrasonic-assisted desilication of parent zeolite, which was synthesized using a rice husk ash as a silica source and various contents of carbon nanotubes (between 0 and 30 wt% of CNTs) playing a role of hard template. It was evidenced that increasing amount of carbon nanotubes in precursor mixture caused the increase of both external surface area and mesopore volume of such prepared ZSM-5-type zeolite product.

In this study, we present an ultrasound-assisted desilication of MFI-type zeolite, which can be used as a catalyst for the dehydration of ethanol into diethyl ether (DEE) and ethylene. DEE can be used in pharmaceutics, explosives and in petrochemistry [33], [34], [35]. In turn, ethylene undergoing polymerisation, oligomerisation, hydrogenation, halogenation, oxidation and a lot of other reactions, has also a great meaning in many industrial processes [36], [37], [38], [39], [40].

In this study, we applied commercial zeolite of MFI type structure (Si/Al = 40) – MFI-40. The physicochemical and catalytic properties of the zeolite-based catalysts prepared in the presence of ultrasounds were compared with those obtained under conventional treatment. Presented research results constitute a precious enrichment of the knowledge concerning the synthesis of hierarchical materials.

## 2 Experimental

### 2.1 Catalyst preparation

The parent MFI-type zeolite (Si/Al = 40) from Zeolyst (CBV 8014) was used as a reference sample. Ultrasonic-assisted desilication was performed using 6 g of zeolite and 200 ml of 0.2 M aqueous solutions of the pure sodium hydroxide (NaOH) or mixture of NaOH and tetrabutylammonium (TBAOH) hydroxide, which contained 10 or 70 mol% of TBAOH (i.e. 0.18 M of NaOH and 0.02 M of TBAOH or 0.06 M of NaOH and 0.14 M of TBAOH, respectively) at the same pH values (13.8).

Alkaline treatment was performed for 30 min. Whole reaction system (alkaline solution, zeolite and the sonicator probe) was placed in an ice bath, which ensured low temperature. QSonica Q700 sonicator with power of 600 W and frequency of 20 kHz was used as a generator of ultrasounds. The device was equipped with a “1” diameter horn (Church Hill Rd, Newtown, CT, USA). For comparison, the conventional desilication (ultrasonic-free procedure) was carried out for 30 min also in the ice bath using alkaline solution at the same chemical compositions as above. In order to investigate a direct influence of ultrasounds on the demineralization intensity, we changed only one parameter, namely, we introduced ultrasonic irradiation into “zeolite-alkaline solution” at the same temperature of ice bath, chemical composition of alkaline solution, mass ratio of zeolite to mixture and the duration of procedure as for conventional demineralization. After desilication procedure, the suspension was fourfold centrifuged at 4000 RPM and dried overnight at 80 °C.

Subsequently, desilicated zeolites were calcined in air with a flow rate of 50 ml/min for 10 h at 525 °C and with a temperature ramp of 1.5 °C/min.

Afterwards, fivefold Na^+^→NH_4_^+^ ion-exchange of desilicated zeolites with 500 ml of 0.5 M aqueous NH_4_NO_3_ solution was performed at 80 °C for 2 h. In next step, the zeolite samples in ammonium form were centrifuged at 4000 RPM, washed, and dried at 80 °C again. Finally, the samples were calcined in 50 ml/min air flow for 8 h at 450 °C and with temperature ramp of 2 °C/min.

The catalysts prepared conventionally or sonochemically were designated by the index “c” or “s”, respectively. Depending on the molar content of TBAOH in 0.2 M NaOH/TBAOH of desilication agent (0 or 10 or 70 mol% of TBAOH), the samples were named as M−0c or M−0 s, M−10c or M−10 s and M−70c or M−70 s, respectively. The parent MFI-type zeolite was denoted as M.

### 2.2 Catalyst characterization

ICP-OES chemical analysis of zeolites was carried out by the dissolution of ca. 100 mg of powder in a HF/HCl mixture in a Teflon vessel for one day. In next step, the liquid was diluted to 250 ml and both Si and Al quantitative analyses were performed using Optima 2100DV - PerkinElmer instrument.

In order to determine the zeolite crystallinity, X-ray diffraction (XRD) experiments were performed using a PANalytical X’Pert PRO MPD diffractometer (40 kV and 30 mA), equipped with CuKα generator (λ = 1.5418 Å). 2θ angle was at 5–50° with a 0.033° step. The zeolite samples were in the form of powder and were placed in holders. The calculations of the average size of crystallites were performed using PANanalytical X Pert Data Viewer software connected with the diffractometer and were based on Scherrer equation (1).

(1) L=λK/βcosθ

Where: ʎ corresponds to X-ray wavelength value (1.5418 Å); K is a dimensionless shape factor (0.9); β means FWHM, i.e. *full width at half maximum* and θ is the Bragg angle.

The status of Al in the investigated samples was determined by the solid-state ^27^Al MAS NMR method using a Bruker Advance III 500 MHz WB spectrometer operating at 11 T of magnetic field. ^27^Al MAS NMR spectra were recorded at 130.3 MHz of the basic resonance frequency and at 10 kHz of a spinning rate (in zirconia rotors) with a short pulse length of 0.2 μs (π/12) and a recycle delay of 0.1 s. 1 M aqueous Al(NO_3_)_3_ was used as a reference for ^27^Al MAS NMR chemical shifts. Prior to NMR experiments, the samples were fully hydrated at ambient temperature in the presence of vapor-saturated Mg(NO_3_)_2_ solution.

The solid-state ^29^Si MAS NMR spectroscopy was used in order to determine the status of silicon. ^29^Si MAS NMR spectra were recorded using a Bruker Advance III 500 MHz WB spectrometer operating at a magnetic field of 11.7 T and at the basic resonance frequency of 99.4 MHz, a spinning rate of 8 kHz (in zirconia rotors) with high-power proton decoupling (SPINAL64), at 5.8 μs (π/3) pulses and repetition time of 20 s. The chemical shifts of ^29^Si MAS NMR spectra were externally referenced to Tetramethylsilane (TMS; >99%).

The porosity was determined by the low temperature sorption of nitrogen at −196 °C using Autosorb-1 Quantachrome. Specific surface area (S_BET_) was determined by BET model, external surface area (S_ext_) and volume of mesopores (V_meso_) were estimated by the application of Barrett-Joyner-Halenda (BJH) model on the adsorption branch of the isotherm. Micropore volume (V_micro_) was calculated using t-plot method. Prior to each measurement, the sample was outgassed for 20 h at 250 °C in a vacuum.

The morphology of the prepared samples was investigated with a JEOL JSM – 7500F Field Emission Scanning Electron Microscope (SEM). Prior to the SEM analysis, the samples were dried for 24 h without the covering of specimens with the coating in order to enable the detailed observation of the surface of the studied materials.

Transmission electron microscopy analysis (TEM) of chosen samples was performed using JEOL JEM 2100 HT LaB6 (JEOL USA, Inc., Peabody, MA, USA), with accelerated voltage of 80 kV and the spot size of 1 nm. Prior to TEM analyses, the studied materials were sprayed onto formvar film coated copper grids.

The FT-IR measurements were conducted with NICOLET iS10 spectrometer (supplied by Thermo Scientific) equipped with a MCT detector. The IR spectra were recorded at 4000–650 ± 4 cm^−1^ with 128 scans per each spectrum. FT-IR measurements were preceded by the activation of samples (in the form of self- supporting wafers of ca. 70 mg) for 1 h at 400 °C with a temperature ramp of 5 °C/min under vacuum conditions.

Quantitative analysis of acidity was performed by the IR studies of the sorption of ammonia (Air Products, 99.95%) at 120 °C and calculated based on the intensities and the extinction coefficients of the bands assigned to ammonia interacting with Brønsted and Lewis acid sites. The bands of 1450 cm^−1^ are attributed to ammonium ions and are characterized by the extinction coefficient of 0.12 cm^2^/μmol, while the maxima at 1620 cm^−1^ correspond to ammonia interacting with Lewis sites with the extinction coefficient of 0.026 cm^2^/μmol [31].

The acid strength of Si-OH-Al groups was determined via CO sorption at −100 °C, followed by the comparison of the values of frequency shifts between the maxima of free acidic OH groups and OH interacting with CO by hydrogen bonding, according to the procedure described in [41].

The dehydration of ethanol into diethyl ether and ethylene as a testing reaction was investigated at 150–290 °C (with a 50 °C step) in a fixed-bed glass microreactor coupled on-line with a gas chromatograph. Analysis of the products obtained in the reaction was carried out in a Perkin Elmer Clarus 580 equipped with Elite-Plot Qcapillary column (with length of 30 m and inner diameter of 0.53 mm) and TCD detector. Prior to each experiment, 100 mg of catalyst (with the granulation of 190–260 μm) was placed on a quartz wool plug in the reactor and exposed to a pure helium flow of 35 ml/min (Air Products, 5.0) at 300 °C for 30 min. Subsequently, He was passed through the liquid ethanol, generating the ethanol in helium flow with a concentration of 2.24 mmol/ml. The total reaction gas mixture stream was 35 ml/min. The weight hourly space velocity was kept at 2.0 g_ethanol_/(g_catalyst_·h).

Turnover frequency (TOF, s^−1^) was defined, as follows:

(2) $$\mathrm{TOF}=n_{Et\left(\mathrm{OH}\right)2}/\left(m\cdot c_{\mathrm{BAS}}\right)$$

Where: n_Et(OH)2_ corresponds to the number of transformed molecules of substrate in one second (µmol/s); m is the catalyst mass (g), and c_BAS_ is the protonic acidity concentration (µmol/g). Experimental error was not higher than 5%.

## 3 Results and discussion

### 3.1 Chemical composition

Analysis of the ICP-OES results (summarized in Table 1) indicated that the treatment of MFI-40 with 0.2 M of NaOH solution in the absence of ultrasounds resulted in a small leaching of both Si (0.5%) and Al (0.1%) from zeolite structure. The addition of ultrasounds caused a significant acceleration in the extraction of both silicon (15.3%) and aluminium (2.6%) from MFI framework, leading to the decrease of Si/Al ratio from 37.7 to 32.4.

**Table 1:** Percentage of leached Si and Al atoms, crystallite sizes and acidity of the synthesized MFI-based catalysts.

Sample	Leaching		Si/Al	Crystallite sizes	Acidic properties		
	Si [%]	Al [%]	ICP-OES	Viewer [Å]	Bronsted [μmol/g]	Lewis [μmol/g]	V_3620_CO…OH [cm^−1^]
M	n.a.	n.a.	37.7	501	228	309	310
M−0c	0.5	0.1	37.5	448	34	53	n.d.
M−0 s	15.3	2.6	32.4	341	70	65	312
M−10c	1.0	1.0	37.5	504	69	63	312
M−10 s	9.4	5.9	36.2	423	53	94	313
M−70c	2.3	2.3	37.6	383	39	105	307
M−70 s	10.8	8.6	36.6	407	47	91	308

In case of the application of demineralizing agent containing TBAOH, the introduction of ultrasonic irradiation into system “zeolite - alkaline mixture” also caused elevated removal of Si and Al from MFI-type zeolite framework. For instance, the use of 0.2 M NaOH/TBAOH solution containing 10% of TBAOH led to the increase of Si and Al extraction from 1.0% to 9.4% and from 1.0% to 5.9%, respectively. Analogous situation was found for the NaOH/TBAOH demineralizing mixture including 70% of TBAOH. The leaching of silicon was 2.3% vs. 10.8%, meanwhile the aluminium extraction was 2.3% vs. 8.6% in the absence vs. presence of ultrasonic irradiation, respectively. The alkaline treatment of MFI structure type zeolites with solutions containing TBAOH led to a slight decrease of Si/Al ratio from 37.7 to 36.2–37.5 due to simultaneous extraction of both elements (Si and Al), although the desilication took place more intensively than dealumination process.

At first sight, it seems that the alkalinity of 0.2 M NaOH should be stronger than 0.2 M (NaOH/TBAOH) due to the presence of TBAOH playing the role of a protective layer on the zeolitic external surface and being a moderator in the desilication process [42], [43], [44]. However, it was found that TBAOH is able to remove aluminum from zeolite framework, which was in line with Sadowska [45] and Abello [46]. This effect rose with the TBAOH mol% content in NaOH/TBAOH demineralizing agent regardless of the way of demineralization procedure (conventional vs. ultrasonic), however, the Al leaching was more intensive under sonochemical conditions. Elevated extraction of aluminum from zeolite framework could lead to the removal of vicinal silicon atoms, which led to the production of holes, followed by the formation of “swiss cheese” type zeolite grains (see TEM analysis, Section 3.4). This effect can be observed only at low temperature (in our case: ice bath conditions). The application of much higher temperature of alkaline treatment always leads to a more intensive desilication, which was reported for the zeolites with MFI [[45], [47], [48], [49], [50]], MTW [50], BEA [51] and FER [52] of similar Si/Al ratio (30–50), for which removal of silicon exceeded 50%. So far, however, a direct introduction of the ultrasonic irradiation into high-temperature desilication performed under identical conditions was not found probably due to technical problems, such as the overheating of sonicator.

In order to avoid the overheating of the source of ultrasounds and investigate a direct influence of ultrasounds on the demineralization intensity, we just introduced ultrasonic irradiation into “zeolite-alkaline solution” at the same (ice bath) temperature, chemical composition of alkaline solution, mass ratio of zeolite to mixture and the duration of procedure like in the case of conventional treatment.

From our ICP-OES results obtained for the desilicated MFI zeolite (Si/Al = 40), it may be concluded that our zeolite was weakly prone to desilication in comparison with FAU-type zeolite of Si/Al = 31 [31]. For faujasite ultrasonically treated with alkaline solutions of various TBAOH content under similar conditions, the percentage amount of Si extracted from framework was 20–40% (for MFI-zeolite was ca 15%). Observed differences in the extraction degree of Si between two types of zeolite structure (FAU vs. MFI) can be explained by a higher stability of MFI zeolite framework than FAU zeolite of elevated Si/Al of 31 (being not natural for the zeolite of this topology, obtained commercially via dealumination of pristine faujasite). Hence, FAU31 zeolite was more sensitive for any modifications (including interaction with aqueous alkaline solutions).

### 3.2 Structure

**Fig. 1:**
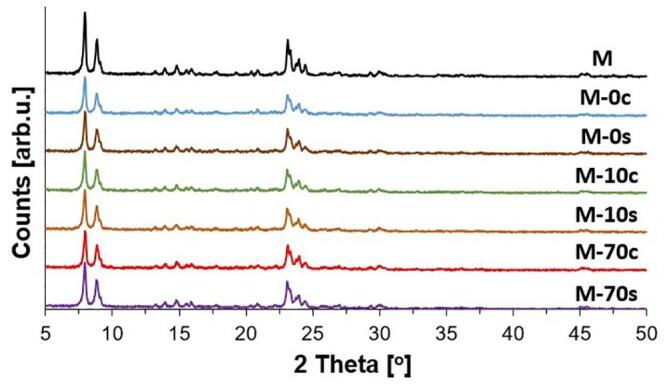
XRD patterns of parent zeolite with MFI structure type (M) and after desilication for 30 min: (c) – classical method, (s) – sonochemically.

The XRD patterns of the investigated catalysts are presented in Fig. 1. For all samples, the occurrence of MFI-type zeolite phase was detected [53]. Analysis of the XRD reflexes of the studied samples leads to the conclusion that neither ultrasonic irradiation nor chemical composition of NaOH/TBAOH alkaline mixture had a significant impact on the crystallinity of the prepared materials. In all cases, the crystallinity was preserved, which well corresponds to the ICP-OES results (Table 1). It may be explained by a limited extraction of both Si and Al from zeolite MFI, which resulted in minor changes of Si/Al of parent material. Hence crystalline structure of modified zeolites did not undergo a collapse.

Based on the analysis of crystallite sizes calculations (Table 1), it can be concluded that the modification of M−40 zeolite with alkaline solutions caused some drops in the crystallite sizes. The average size of crystallites decreased from 501 to 341–448 Å. Registered changes in the size of crystallites did not reveal apparent relationship with the conditions of alkaline treatment procedure.

Abello et al. [46], Schmidt et al. [47], Groen et al. [54], Rutkowska et al. [55], [56], [57] and Ahmadpour et al. [58] also did not observed significant changes in crystalline structure of desilicated MFI-type zeolites in respect to parent samples of similar Si/Al ratio.

**Fig. 2:**
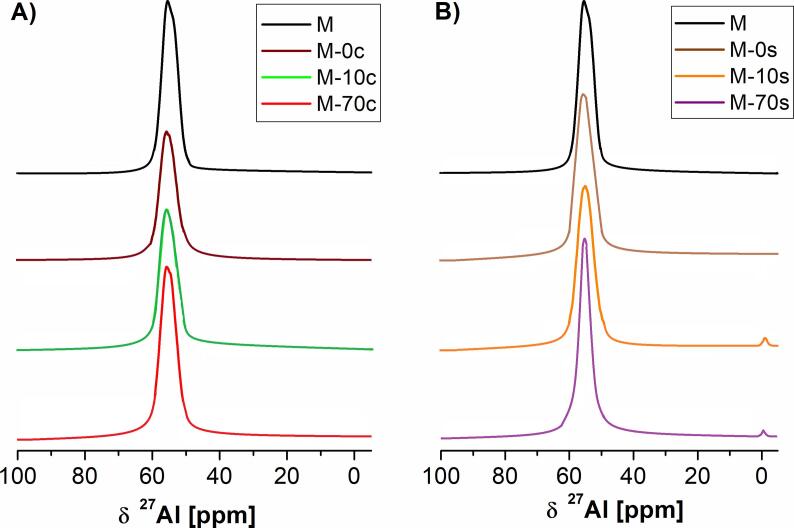
^27^Al MAS NMR spectra of the studied samples prepared via: A) conventional B) sonochemical route using alkaline solutions (NaOH/TBAOH) containing 0, 10 or 70% mol of TBAOH.

^27^Al MAS NMR spectra (Fig. 2) illustrate the status of Al in the prepared catalysts. For the reference sample (M), the occurrence of the Al signal at ca 57 ppm is attributed to zeolite framework tetrahedral aluminum [45], [50]. Simultaneously, very weak signal at 0 ppm originating from extra-framework octahedral Al species was found. The status of Al depended slightly on both the application of ultrasounds during desilication and the chemical composition of 0.2 M NaOH/TBAOH aqueous solution. In the absence of ultrasounds, aluminum did not go from tetrahedral to octahedral positions. According to Sadowska et al. [45], the treatment of zeolite MFI with NaOH did not lead to the formation of extra-framework aluminum species, what means that mild desilication allows to stay all Al atoms in zeolite framework. The lack of the growth of signal at 0 ppm can be also explained by the reinsertion of Al into the zeolite framework (known as realumination), which was previously reported for zeolites with FAU [41], [59], [60] or MFI-type structure [[45], [54], [61], [62]]. In case of ultrasonic-assisted procedure of demineralization of MFI-40 zeolite with alkaline mixture containing TBAOH, the removal of aluminum from tetrahedral coordination is accompanied by a slight formation of extra-framework Al species.

**Fig. 3:**
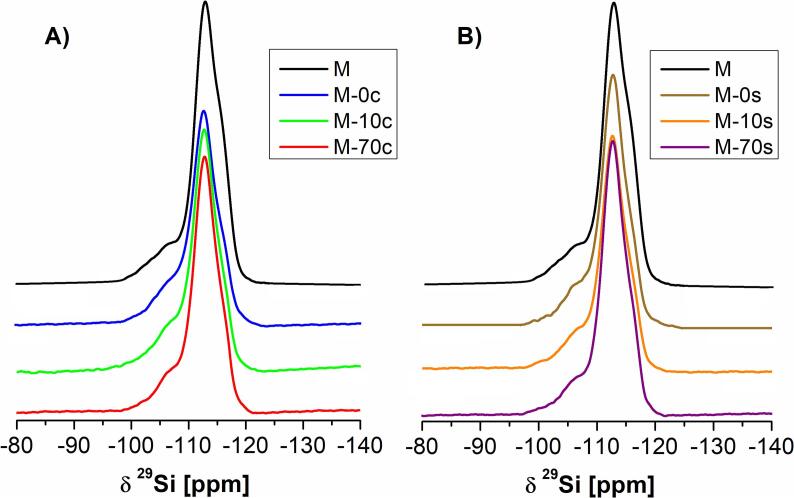
^29^Si MAS NMR spectra of the studied samples prepared via: A) conventional B) sonochemical route using alkaline solutions (NaOH/TBAOH) containing 0, 10 or 70% mol of TBAOH.

Fig. 3 shows the ^29^Si MAS NMR spectra of the prepared samples. In all cases, the occurrence of the Si signals at −112 and at −108 ppm originates from Si(4Si) and Si(3Si), respectively [45], [50]. The Si(3Si) signal correspond mainly to Si(1Al) surroundings. The Si(4Si) signal was dominating for all investigated catalysts. For all studied samples, the ^29^Si MAS NMR spectra given in Fig. 3 and data obtained from the deconvolution of ^29^Si MAS NMR spectra (Table S1) are similar. Generally, the status of silicon in the studied samples was independent of the route of demineralization (conventional vs. ultrasonic) and chemical composition of the alkaline mixture applied during the procedure of modification. Slight differences in the intensity of Si(4Si) signals correspond to the leaching of Si (and to a lesser content of Al), resulting in slight changes of Si/Al ratios (Table 1). In both series (conventional vs. ultrasonic-assisted), the most intensive Si(4Si) signals were found for the zeolites modified with the NaOH/TBAOH with TBAOH content of 70 mol% due to the highest Al extraction (and Si/Al ratio). Minor changes in the status of either Si and Al also agree with the crystallinity of the prepared samples (Fig. 1).

### 3.3 Porosity

The results of the porosity of the prepared materials are summarized in Table 2 and Figure S1. The parent MFI zeolite is characterized by the presence of both micropores (0.162 cm^3^/g) and intercrystalline mesopores (0.185 cm^3^/g) with the average pore diameter of 30.3 Å. Hence, the percentage contribution of mesopores volume was 53.3%.

**Table 2:** The impact of the modification procedure route on the porosity of the synthesized MFI-based catalysts.

Sample	S_BET_ [m^2^/g]	(S_ext_/S_BET_)*100% [%]	V_total_ [cm^3^/g]	(V_meso_/(V_total_)*100% [%]	D [Å]
M	458	16.6	0.347	53.3	30.3
M−0c	231	18.2	0.215	61.9	37.3
M−0 s	324	20.7	0.340	67.6	42.1
M−10c	301	19.3	0.282	62.8	37.5
M−10 s	335	22.4	0.315	64.8	37.7
M−70c	328	17.4	0.319	63.0	38.9
M−70 s	398	20.1	0.415	66.7	41.7

The way of the alkaline treatment influenced the porous structure of the prepared catalysts. The modification of MFI-40 with 0.2 M NaOH under conventional conditions led to a decrease of the total volume of pores from 0.347 to 0.215 cm^3^/g with simultaneous growth of the mesoporosity to 61.9%. That caused growth of the average pore diameter from 30.3 to 37.3 Å.

The use of the ultrasonic-assisted technique led to more significant production of mesoporosity in relation with the conventional method of modification of MFI-40. The volume of micropores decreased from 0.162 to 0.110 cm^3^/g and the volume of mesopores and average pore diameter rose from 0.185 to 0.230 cm^3^/g and from 30.3 to 42.1 Å, respectively. That led to the increase of percentage mesoporosity contribution up to 67.6%.

The appearance and further rising of TBAOH in the demineralising agent strengthened the formation of mesoporosity in prepared catalysts. Furthermore, all zeolite samples modified sonochemically revealed higher mesoporosity than counterparts prepared classically. The use of 0.2 M NaOH/TBAOH (10 mol% of TBAOH) in the absence or presence of ultrasounds caused the increase of mesopores volume participation from 53.3% to 62.8% vs. 64.8% and the increase of average pore diameter from 30.3 to 37.5 vs. 37.7 Å, respectively. In the case of the utilization of the NaOH/TBAOH alkaline solution containing 70 mol% of TBAOH, a minimal increase of mesoporosity from 53.3% to 63.0% vs. 66.7% with a simultaneous growth of an average pore diameter from 30.3 to 38.9 vs. 41.7 Å were found.

It also was found that an alkaline treatment of MFI-40 zeolite resulted in a minimal growth of percentage S_ext_/S_BET_ ratio from 16.6% to 17.4–22.4%. However, all catalysts prepared ultrasonically demonstrated higher S_ext_/S_BET_ values (20.1–22.4%) in comparison with the samples modified conventionally (17.4–19.3%).

For all studied samples, the appearance of hysteresis loop of IV type can be attributed to the presence of both intercrystalline pores between the MFI crystals (particularly for parent sample “M”) and the formation of mesoporosity.

According to the XRD patterns illustrated in Fig. 1 as well as ICP-OES data summarized in Table 1, small changes in the crystallinity are in line with minor changes in porous structure of the studied samples.

Our results are in good agreement with, Zhang et al. [29], Oruji et al. [30], Khoshbin et al. [32] as well as with our previous studies [31]. It was evidenced that a sonochemical demineralization procedure enhanced the production of higher mesoporosity in respect to the conventional alkaline-treatment technique. Additionally, it was indicated that the use of ultrasounds during modification resulted in preserved microporosity. On the other hand, observed changes in the porous structure of our MFI-type zeolites are significantly smaller than for MFI-type analogues desilicated by Sadowska et al. [45], [49], Abello et al. [46], Shmidt et al. [47], Gil et al. [50] and Groen et al. [62] due to the use of much milder conditions in our experiments (relatively short duration of the procedure, ice bath temperature, and respectively low concentration of desilicating agents) in comparison with the research quoted above.

### 3.4 Morphology

**Fig. 4:**
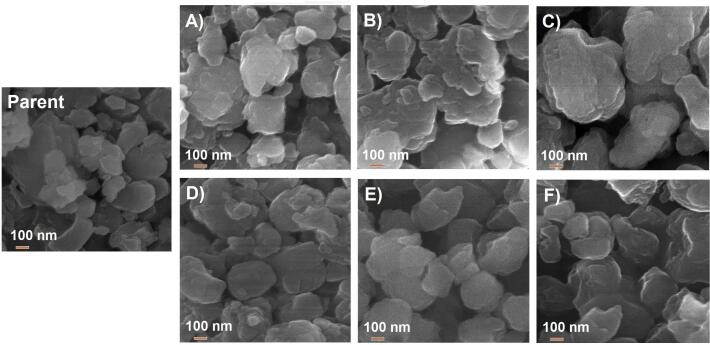
Morphology of the prepared catalysts (x50k): parent, A) M−0c, B) M−10c, C) M−70c, D) M−0 s, E) M−10 s and F) M−70 s.

The analysis of the morphology of the prepared MFI-40-based catalysts are illustrated in Fig. 4 (magnification to 50,000x). The appearance of SEM images leads to the conclusion that the particles of all modified samples are of irregular shape with dimensions ranging from 300 to 1000 nm. For the samples treated under conventional conditions (Fig. 4a-c), alkaline treatment led to some fragmentation of grains, which slightly influenced the porosity of this series of samples.

In case of the catalysts prepared via ultrasonic-assisted procedure (Fig. 4d-f), treatment of the parent zeolite (M) led to the appearance of cracks, cavities and strengthened the fragmentation of grains in comparison with the analogues modified in the absence of ultrasonic irradiation. Observed changes in microscopy well correlate with crystallite sizes and textural properties given in Table 2. Nevertheless, the registered changes in the morphology of the investigated MFI-type zeolite materials are quite small, particularly when we compare our current samples (MFI-40) with the materials based on FAU-31 zeolite, which we reported in [31]. Relatively stable morphology of our MFI-type catalysts is in line with their preserved crystallinity (Fig. 1).

Furthermore, from analysis of the SEM pictures, it may be concluded that the chemical composition of demineralizing mixture (meant as NaOH/TBAOH ratio) both with and without ultrasounds had no apparent impact on the size and the shape of zeolite crystals.

**Fig. 5:**
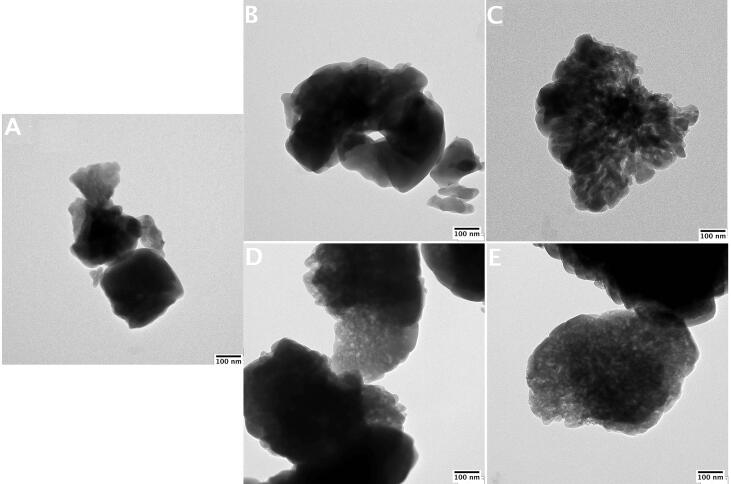
TEM images of the prepared catalysts (x50k): A) parent, B) M−0c, C) M−0 s, D) M−70c and E) M−70 s.

For comparison, the TEM images (50,000×) of the variously prepared catalysts are illustrated in Fig. 5. Analysis of the appearance of crystalline grains led to the conclusion that alkaline treatment of the parent M−40 zeolite caused the production of holes and the formation of “swiss cheese”-type zeolite grains followed by the changes in porous structure of the prepared MFI-based samples. Observed perforation seems to be more apparent in the case of zeolites desilicated in the presence of ultrasounds (Fig. 5 C and E). Nevertheless, observed changes in the appearance of zeolite grains were not very sharp, which was in line with crystallinity, structure, morphology and subtle changes in porosity of the studied samples.

### 3.5 Acidity

**Fig. 6:**
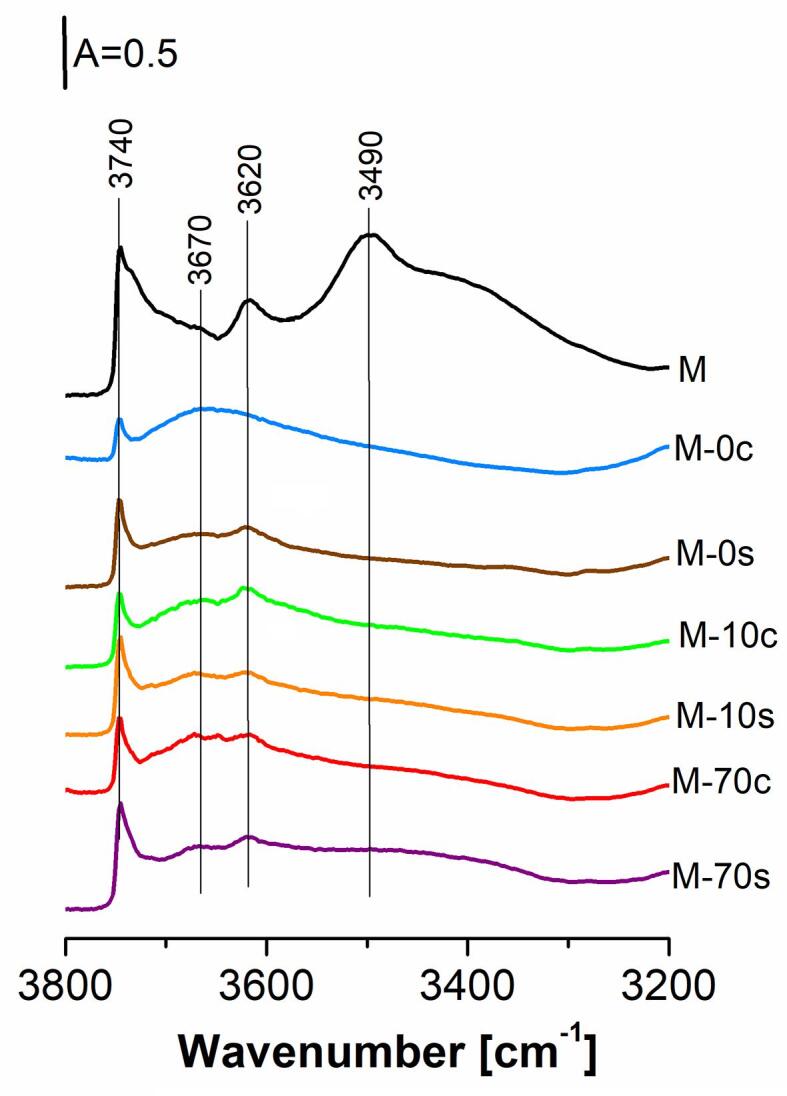
IR spectra of OH groups region recorded for parent MFI zeolite (M) and after alkaline treatment using: (c) – conventional method, (s) – ultrasonic assisted procedure.

The characterization of the acidity of the prepared MFI-type samples was illustrated in Fig. 6 and Table 1. For the parent sample (M), the IR spectrum of the OH groups region indicated the presence of the bands at: 3740 cm^−1^ attributed to external Si-OH groups with a shoulder at 3730 cm^−1^ of silanols in the defects, 3670 cm^−1^ assigned to Al-OH, 3620 cm^−1^ originating from acidic Si-OH-Al groups and at 3490 cm^−1^ coming from silanol nests [45], [49], [50], [62].

Alkaline treatment with 0.2 M NaOH or NaOH/TBAOH aqueous solutions led to significant changes in the appearance of IR spectra. The disappearance of the band at 3490 cm^−1^ as well as an apparent decrease of the signals at 3740 and 3620 cm^−1^ demonstrated the removal of a significant part of OH groups during desilication procedure. Another effect was a slight increase of the band at 3670 cm^−1^, which suggests that the interaction of MFI-40 zeolite with alkaline mixtures caused the production of Al-OH groups.

Information on the strength of the acidic OH groups (in Si-OH-Al) was given in Table 1. The data obtained from CO sorption showed that the modification of parent MFI-40 zeolite with NaOH or NaOH/TBAOH mixtures both in the absence or presence of ultrasounds did not influence the acid strength of Si-OH-Al (Δν_3620_OH…CO = ca 310 cm^−1^). In case of M−40 zeolite treated with NaOH solution under conventional conditions (M−0c), the broad band with the maximum at 3670 cm^−1^ probably overlapped with the band at 3620 cm^−1^ of Si-OH-Al, making the latter signal undetectable, therefore the strength of protonic acid sites was not determined for M−0c.

Our results obtained in the current study are in contrast to other research reported for zeolites with MFI [49] and BEA [63], for which desilication caused the decrease of the acid strength of Si-OH-Al by ca. 10–20 cm^−1^, which derived from the occurrence of the more and less acidic OH groups. This contradiction may be explained by the fact that our MFI-40 zeolite was modified under milder conditions in comparison with the majority of experimental procedures reported in the available literature. Furthermore, taking into account relative high amounts of Al extracted from zeolite structure in respect to Si, it could be possible that the process of demineralization of our MFI-40 zeolite caused the destruction of Si-OH-Al groups of the highest and the lowest acid strength. Alternative situation might refer to the removal of Si and Al responsible for Si-OH-Al of medium acid strength. Hence in both suggested cases, an average acid strength of the remaining protonic acid sites after MFI-40 treatment under presented conditions did not undergo apparent changes.

The results on the quantitative analysis of the acid sites present in the prepared catalysts are given in Table 1. In all cases, the modification of the parent MFI-40 zeolite with NaOH or NaOH/TBAOH mixtures caused a strong decrease of both Brønsted and Lewis acid sites from 228 to 34–70 μmol and from 309 to 53–105 μmol/g, respectively. The observed tendency implies a simultaneous removal of silicon and aluminum from modified MFI-type zeolite. It also was found that the technique of alkaline treatment slightly influenced concentrations of both types of acid sites. Generally, the application of ultrasonic-assisted procedure of MFI-40 modification with the alkaline mixture at concrete chemical composition resulted in a little higher concentrations of acid sites than for counterparts prepared conventionally. Obtained data agrees with the results of ICP-OES analysis, which showed that the presence of ultrasounds during modification procedure of MFI-40 zeolite shifted demineralization towards Si extraction from zeolite framework. For comparison, the interaction between MFI-40 zeolite and alkaline mixture under ultrasonic-free conditions caused practically equal Si and Al removal from zeolite structure (Table 1).

### 3.6 Catalytic properties

**Fig. 7:**
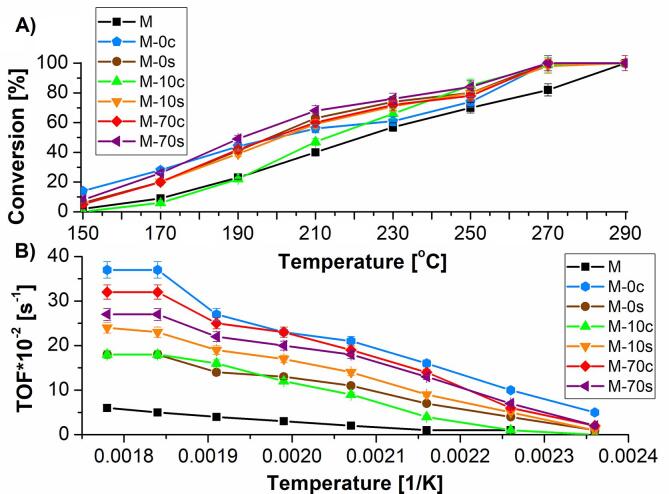
A) Conversion of ethanol at 150–290 °C and B) Turnover frequency in the dehydration of ethanol over MFI type zeolite - based catalysts.

Catalytic activity of variously prepared MFI-type zeolite - based catalysts in the dehydration of ethanol reaction was depicted in Fig. 7A.

For all studied catalysts, It was found that catalytic activity rose with the temperature of experiment in a whole range. In the case of a reference catalyst (M), at 150 °C, 170 °C, 190 °C, 210 °C, 230 °C, 250 °C 270 °C and 290 °C, the conversion of ethanol formed the sequence, as follows: 2%, 9%, 23%, 40%, 57%, 70%, 82% and 100%, respectively. It also was indicated that an alkaline treatment of MFI-40 zeolite (independently of the procedure conditions) led to the enhancement of the catalytic properties of the prepared materials. For all zeolites treated with basic solutions, the conversion of ethanol was 100% at 270–290 °C. The best results were found for the catalyst modified under ultrasonic-assisted conditions with NaOH/TBAOH solution (containing 70 mol% of TBAOH (M−70 s), for which the conversion of ethanol was 8%, 26%, 49%, 68%, 76%, and 84% at 150 °C, 170 °C, 190 °C, 210 °C, 230 °C and 250 °C, respectively. For comparison, some worse conversion of ethanol was found for the counterpart prepared in the absence of ultrasounds (M−70c), which was 5%, 20%, 42%, 60%, 72% and 78% at the same temperature range, as above.

The presence of ultrasonic irradiation during alkaline treatment of MFI-40 type zeolites also improved catalytic activity in the case of the NaOH/TBAOH solutions containing 10 mol% of TBAOH. At 150 °C, 170 °C, 190 °C, 210 °C, 230 °C and 250 °C, the conversion of ethanol was 0% vs. 5%, 6% vs. 20%, 22% vs. 39%, 47% vs. 59%, 65% vs. 71%, 80% vs. 85% for the catalysts prepared conventionally and sonochemically, respectively.

Much weaker effect of the application of ultrasounds during demineralization procedure of zeolites on their catalytic activity was found for the samples modified with NaOH solution. At 150–190 °C, the conversion of ethanol was higher for the catalysts prepared classically (14%-44% for M−0c vs. 6%-41% for M−0 s), while the zeolites treated ultrasonically revealed higher catalytic activity at 210–250 °C (56%-74% for M−0c vs. 63%-80% for M−0 s).

The observed effects correspond to the degree of demineralization of zeolites and the production of mesopores, which facilitated transport of the reagents within porous structure of investigated catalysts.

Alkaline treatment of MFI-40 zeolite also influenced the reaction rates in the prepared catalysts (Fig. 7B). Analysis of turnover frequency (TOF) results led to the conclusion that the modification of the parent zeolite (M) with alkaline solutions raised TOF values, which agreed with the changes in either porous structure or acidity of the prepared catalysts (Table 1, Table 2) and with the results reported by Verboekend and Pérez-Ramírez [42].

It also was found that the application of ultrasonic irradiation during preparation procedure of catalysts (M−0 s and M−70 s) resulted in lower TOF values than for the analogues prepared conventionally (M−0c and M−70c). An opposite tendency was indicated for M−10c and M−10 s. Observed correlations between TOF values implied from catalytic activity as well as from concentrations of protonic acid sites (responsible for this reaction). Actually, the catalysts prepared ultrasonically revealed generally higher conversion of ethanol, but the concentrations of protonic acid sites also were higher for this group of materials (with one exception for M−10 s) in comparison with the catalysts modified conventionally. Direct comparison of the changes in the concentration of Brønsted acid sites (Table 1) and catalytic activity (Fig. 7A) allows us to claim that a quantitative analysis of active centres has a stronger impact of TOF values than the conversion of ethanol at given temperatures.

**Fig. 8:**
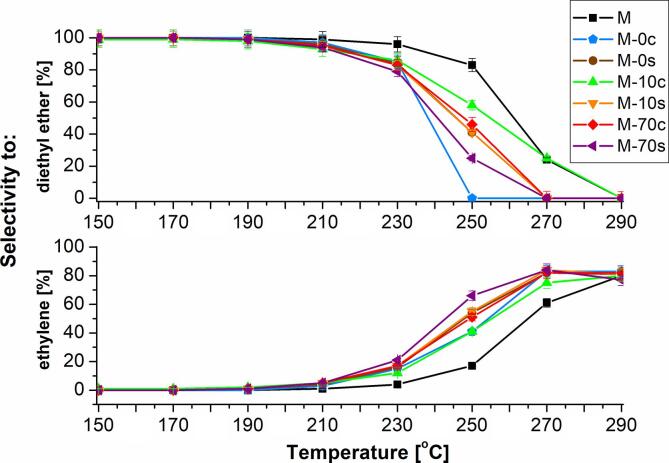
Selectivity to A) diethyl ether or B) ethylene at 150–290 °C on prepared catalysts.

Analysis of the results obtained from catalytic performance illustrated in Fig. 8A and 8B indicated clearly that both the type and amount of a concrete product is determined by the temperature range. Up to 210 °C, it was possible to manufacture practically pure diethyl ether: for all studied catalysts, selectivity was 93–100%. At higher temperatures, the appearance of ethylene was registered. It is worth to underline that no pure ethylene was produced at 210–290 °C due to both co-existence of diethyl ether (at 210–270 °C) and the coking occurring at the highest temperatures of experiment.

Significant selectivity to ethylene was found for all studied catalysts at 250–290 °C. For parent zeolite (M) it was 17–80%. Alkaline treatment of MFI-type zeolites resulted in a visible enhancement of selectivity to ethylene, however, this effect was generally stronger for the samples after ultrasonic irradiation. For instance, in case of MFI-type zeolite sonochemically modified with NaOH/TBAOH alkaline solution containing 10 mol% of TBAOH (M−10 s), selectivity to ethylene was 55–84%, meanwhile for zeolite modified conventionally (M−10c), selectivity to ethylene was 41–80%. Other by-products like acetaldehyde were not detected.

The best catalyst was M-70s, for which the highest conversion of ethanol was found. Additionally, M-70s demonstrated very high selectivity to diethyl ether (94-100%) at 150-210 °C and the highest selectivity to ethylene among investigated catalysts (21%, 66% and 84%) at 230 °C, 250 °C and 270 °C.

Similar observations were reported by Oliveira [64], who investigated the ethanol dehydration on Cu- and Fe-ZSM-5 catalysts. The production of diethyl ether (70–100%) was found in the temperature range of 180–200 °C on Cu-ZSM-5, while ethylene was formed mainly at temperatures exceeding 200 °C over both pure and Fe containing ZSM-5 catalysts (20–100% depending on the type of catalyst). For comparison, Zhan et al. [65] reported yield of DEE reaching 67% using 2% PHZSM-5 catalyst. In turn, Jinfa et al. [66] obtained yield of DEE exceeding 70% at 180 °C over ZSM-5.

Our catalytic results are also in line with Phung, Chiang et al. [[33], [67], [68], [69]], who studied the ethanol dehydration on commercial H-FER, H-MFI, H-MOR, H-BEA, H-Y and H-USY zeolites For the investigated systems, diethyl ether was mainly produced at low temperatures, while the production of ethylene was predominant at high temperatures, which was independent of the type of acid sites of the studied catalysts. It was found that at 180 °C, the selectivity to DEE was higher for H-MFI and H-BEA (exceeding 70%) than for other zeolites. On the other hand, at high temperature, almost full selectivity to ethylene was registered for H-FER, H-Y and H-USY, while co-production of higher hydrocarbons took place in the case of H-MFI, H-BEA and H-MOR, which was in line with the data reported by Stepanov et al. [70] and Wang et al. [71]. Rising reaction temperature led to the formation of coke, particularly over H-MOR and H-BEA. The strength of protonic acid sites was found as similar for all studied zeolites, which was in agreement with Xu et al. [72] and did not correlate with catalytic activity and selectivity.

It was also shown [[33], [67], [68], [69]] that porous structure and morphology of the investigated catalysts influenced the catalytic properties in given reaction. Medium pore zeolites, such as H-MFI, H-BEA and H-MOR demonstrated the highest selectivity to diethyl ether (98%) at moderate temperatures (180 °C). It may be concluded that zeolites of medium size channels are the most suitable for this reaction than counterparts possessing either larger or smaller cavities (faujasites or ferrierite, respectively). That implies from a confinement effect favouring the production of diethyl ether at lower temperatures and the shape selectivity enabling the formation of ethylene at higher temperatures. It may be also explained by the different kinetic behaviour of these two reactions, namely, in higher activation energy and lower ethanol reaction order towards ethylene formation in relation to DEE.

Almost full selectivity to ethylene and yield were obtained at high temperature over small-pore H-FER and on large-pore H-Y and H-USY [69]. In the case of medium-pore zeolites like H-MFI, H-MOR and H-BEA, the selectivity to ethylene was limited by the production of higher hydrocarbons and coke.

Detailed mechanism of ethanol dehydration on protonic form of zeolites with MOR, MFI and FER type structures was described by Phung, Chiang et al. [[33], [67]]. It was explained that a selective production of diethyl ether from ethanol occurs at lower temperature by the reaction of ethoxy groups with undissociated ethanol. At higher temperatures, the formation of ethylene is going via decomposition of ethoxy groups on catalysts containing active acid sites.

Osuga et al. [73] also investigated ethanol dehydration over MFI- and MOR-type zeolites and under different contact time conditions. DEE was found as one of the initial reaction products, influencing the catalytic activity. At the same catalyst weight / ethanol flow ratio (24.3 g·h/mol), for MFI zeolite, DEE was detected as dominating product with selectivity reaching 100%, whereas ethene was produced over mordenite with selectivity reaching 60%. Therefore, it may be concluded that the dehydration of ethanol over zeolites with two different structure types can occur via different reaction routes.

According to Madeira et al. [74], both the acidity and porous structure of zeolites determined their catalytic behaviour in the ethanol conversion into hydrocarbons possessing three carbon atoms or more (C_3+_). It was indicated that high pore size H-FAU and H-BEA type zeolites were characterized by a high yield of ethylene and diethyl ether as well as low contribution of C_3+_ hydrocarbons in comparison with medium pore zeolite HZSM-5. This observation can be explained by a faster deactivation of large pore zeolites, which was caused by the formation of coke, eliminating protonic acidity, responsible for the transformation of ethylene into higher hydrocarbons. For H-ZSM-5 zeolite, after 16 h of reaction, practically full conversion of ethanol towards C_3+_ hydrocarbons (including butenes, parafins and aromatics) was found. In turn, in the case of large pore zeolites (H-BEA, H-FAU), high amounts of by-products like more condensed aromatics were detected. Furthermore, it was found that for H-ZSM-5 zeolite, the deactivation was slower and the production of C_3+_ hydrocarbons was found even under the saturation of the catalyst with coke molecules, thus it may be supposed that for this zeolite, reaction could take place at the pore mouth of the channel.

According to Däumer et al.[75], the formation of C_3+_ hydrocarbons is favoured in the zeolites of relatively narrow cavities, thus the acid sites strength present in micropores plays a supporting role in the production of higher hydrocarbons. It was also reported that the formation of C–C bonds was strongly dependent on the presence of highly strong Brønsted acid sites. In turn, the intermediate species stability in zeolite framework depended on interaction between fragments of confined hydrocarbon and zeolite framework [76]. The size of cavities influences the stability of the confined species defined by an electrostatic and van der Waals interaction between fragments of hydrocarbons and zeolite framework.

Our findings are also in line with Gołąbek et al. [77], who investigated the role of pore arrangement of 10-ring zeolites (ZSM-5, TNU-9 and IM-5) on their catalytic properties in ethanol transformation. From obtained data, it was concluded that all studied catalysts were active at 150–300 °C and at atmospheric pressure, leading to the production of diethyl ether (DEE) and ethylene as the products. It was also shown that the conversion of ethanol increased with reaction temperature. ZSM-5-based catalysts did not undergo deactivation and small and uniform ZSM-5 crystals did not affect catalytic lifetime.

Based on available literature, a beneficial impact of the application of ultrasonic irradiation in the synthesis of zeolite-based materials on their physicochemical and catalytic properties has been reported. For instance, Oruji et al. [30] performed ultrasonic-assisted desilication of NaY zeolite in order to prepare hierarchical materials with elevated mesoporosity and higher crystallinity than zeolites treated conventionally. Independently of the used technique, the mesoporosity of desilicated FAU-type zeolite gradually increased with the duration of procedure and was higher for the samples modified sonochemically. In the reaction of catalytic cracking of middle distillate at 550 °C, the it was revealed that all sonochemically treated samples demonstrated higher catalytic activity with their high liquid and low gas yields (78–86% and 14–22%, respectively). Coking was practically absent. Furthermore, it was shown that the catalyst lifetime for sonochemically prepared materials was higher than for the samples treated conventionally due to more apparent destruction of microporosity with simultaneous more noticeable generation of mesoporosity in zeolite.

Another example of beneficial influence of ultrasounds during catalysts preparation is an ultrasonic-assisted deposition of active phase on zeolite carrier, which was reported in our previous papers [78], [79], [80]. Jodłowski et al. [78] reported that sonochemically prepared structured reactors with a deposited copper on ZSM-5 and USY zeolite revealed a full NO conversion and almost constant 100% selectivity to nitrogen in SCR- DeNO_x_ reaction. In turn, Chlebda et al. [79] indicated that ultrasonic procedure of iron containing ZSM-5-based catalysts preparation enhanced catalytic activity in the DeNO_x_ process, with almost full selectivity to N_2_. Sobuś et al. [80] reported that Cu- and Co containing BEA zeolites revealed the best catalytic properties in the Selective Conversion of Lactic Acid into Acrylic Acid reaction with the selectivity to Acrylic Acid exceeding 60%.

## 4 Summary and conclusions

In this research, we investigated an ultrasonic-assisted desilication of zeolite with MFI type structure using aqueous NaOH/TBAOH alkaline solutions of various chemical compositions. Subsequently, we compared the physicochemical and catalytic properties of such prepared samples with counterparts desilicated in the absence of ultrasounds.

It was shown that the application of ultrasounds during demineralization procedure caused higher both silicon and aluminum extraction in comparison with analogues treated classically. The Si and Al contents leached from MFI-type zeolite framework were in the range of 0.5–15.3% and 0.1–8.6%, respectively. Si/Al ratio of the modified MFI-based samples was in the range of 32.4–37.6 and was only slightly lower in respect to the reference sample (37.7). Alkaline treatment of MFI-type zeolite resulted in the formation of “swiss cheese” - type zeolite grains containing numerous holes inside zeolite crystallites, which was more visible for the zeolites modified under ultrasonic-assisted procedure.

Surprisingly, we observed that the conducting of demineralization procedure (independently of the presence/absence of ultrasonic irradiation) did not alter the crystallinity, structure and morphology of the modified materials. Nevertheless, the application of ultrasounds during alkaline treatment procedure led to the production of higher mesoporosity, which enabled better mass transfer of reagents in porous structure and therefore caused enhanced catalytic properties in the reaction of the dehydration of ethanol in relation to the catalysts obtained under conventional demineralization conditions.

Furthermore, it was indicated that independently of the alkaline treatment technique (conventional vs. ultrasonic), a notable decrease of both protonic and Lewis acidity corresponded to a simultaneous leaching of Si and Al from the structure of MFI-type zeolite.

The analysis of the results presented above led to the conclusion that ultrasonic assisted demineralization of MFI-type zeolite led to the production of attractive catalysts with easily accessible active sites for the ethanol processing.

## CRediT authorship contribution statement

**Ł. Kuterasiński:** Conceptualization, Data curation, Formal analysis, Methodology, Software, Validation, Supervision, Visualization, Writing - original draft, Writing - review & editing. **U. Filek:** Formal analysis, Investigation, Methodology. **M. Gackowski:** Formal analysis, Investigation, Methodology, Visualization, Writing - review & editing. **M. Zimowska:** Formal analysis, Investigation, Methodology. **M. Ruggiero-Mikołajczyk:** Formal analysis, Investigation, Methodology. **P.J. Jodłowski:** Formal analysis, Investigation, Methodology, Visualization, Writing - review & editing.

## Declaration of Competing Interest

The authors declare that they have no known competing financial interests or personal relationships that could have appeared to influence the work reported in this paper.
