# Supplementary material for: Sonochemically prepared hierarchical MFI-type zeolites as active catalysts for catalytic ethanol dehydration
Source: Ultrason Sonochem. 2021 May 3;74:105581. doi: 10.1016/j.ultsonch.2021.105581 (PMC8129989; doi:10.1016/j.ultsonch.2021.105581)
Supplement: Supplementary data 1 [file mmc1.docx]

**SUPPLEMENTARY MATERIALS**

Table S1. Quantitative analysis of different surroundings of silicon taken from deconvolution of ^29^Si MAS NMR spectra of prepared materials. Percentage values come from contributions of the signal areas of different Si surroundings.

| **Sample** | **Si(0Al)** | **Si(1Al)** | **Si(2Al)** |
| --- | --- | --- | --- |
| **M** | 82% | 11% | 7% |
| **M-0c** | 72% | 22% | 6% |
| **M-0s** | 76% | 19% | 5% |
| **M-10c** | 77% | 20% | 4% |
| **M-10s** | 76% | 18% | 5% |
| **M-70c** | 77% | 18% | 5% |
| **M-70s** | 81% | 15% | 4% |


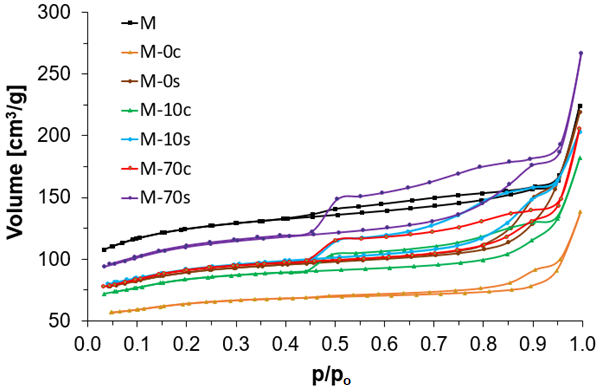


Figure S1. Adsorption-desorption isotherms of the nitrogen at -196 ^o^C for the studied samples.
